# Genomic Evidence for Local Adaptation to Elevation and Climate Sheds New Light on Variable Responses to Global Change in American Pikas ( *Ochotona princeps* )

**DOI:** 10.1111/mec.70238

**Published:** 2026-01-18

**Authors:** Erik A. Beever, Elizabeth Osterhoudt, Ethan B. Linck

**Affiliations:** ^1^ U.S. Geological Survey Northern Rocky Mountain Science Center Bozeman Montana USA; ^2^ Department of Ecology Montana State University Bozeman Montana USA

**Keywords:** adaptation, genomics/proteomics, global change and drought, mammals

Local adaptation is a key mechanism permitting species to accommodate diverse or changing environmental conditions across their geographic range (Meek et al. 2023). Intraspecific adaptive variation complements phenotypic plasticity (e.g., in diel activity schedules, diet components, use of microrefugia), being eurytopic (i.e., having wide niche breadths), and dispersal as fundamental alternative strategies to cope with challenging conditions (Thurman et al. 2020). Home to largely intact natural landscapes and sharp abiotic gradients connecting diverse habitats and biotas, mountains are powerful laboratories to test a wealth of eco‐evolutionary hypotheses that also have implications for management, conservation and climate adaptation. Few taxa are as closely associated with these inspiring, threatened landscapes as American pikas ( *Ochotona princeps* ). As Farrand et al. (2025) note, the generally philopatric tendencies of the species, the spatially structured nature of its broken‐rock habitats (Figure 1a), and its geographically vast, environmentally variable range provide a magnificent canvas for investigating drivers and patterns of local adaptation. The authors take full advantage of this canvas in their work, identifying geographically restricted genetic variants associated with hypoxia, severe cold, and dietary toxins.

**FIGURE 1:**
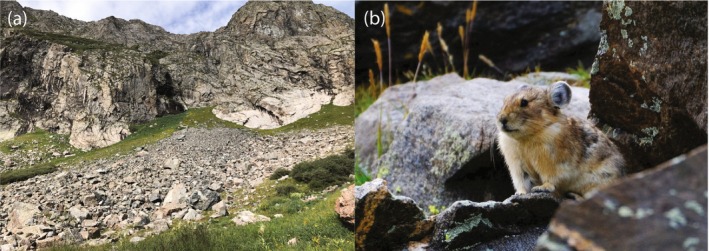
Examples of the article's study species and habitat. (a) Example of American pika *(Ochotona princeps)* habitat in southern Colorado, photo credit: Zachery Farrand; (b) an American pika, photo credit: Matt Betts.

To do so, Farrand et al. capitalise on what is by far the most extensive and comprehensive collection of high‐quality tissue samples from *O. princeps* (*n* = 161 specimens from an original 64 sites; Galbreath et al. 2010). Their elegant research leverages a published reference genome and several earlier studies of genetic variation in *O. princeps* in combination with decades of research on the species' demography, behaviour, distribution, nutritional ecology, and ecophysiology. The team knows the system well: earlier work by this paper's second author led to revision of the taxonomy of *O. princeps* , reducing the number of recognised subspecies from 36 (based on traditional cladistics and morphometrics; Hall 1981) to 5 reciprocally monophyletic lineages (based on phylogenies estimated with both nuclear and mitochondrial DNA; Galbreath et al. 2009). Although these lineages (plus a sixth lineage identified subsequently) and their relationships inform our understanding of genome‐wide population divergence, the role of adaptive processes in shaping pika evolution has remained more poorly known.

Understanding local adaptation in *O. princeps* is especially important for explaining and predicting variable responses to environmental change across its range. *O. princeps* is noteworthy in having (1) some of the lowest published values for observed heterozygosity among mammals, hinting at limited adaptive potential (Beever et al. 2023, supplemental information); (2) patterns in abundance, occupancy, dispersal, physiology, and genetics that are strongly predicted by weather or climate across spatial and temporal scales; (3) variable responses to environmental change across ecoregions (Smith et al. 2019); and (4) sharp, widespread population declines despite small body size, stable habitat area, no appreciable harvest or persecution, and persistently high densities in some localities. Together, these observations suggest pika demography is likely to be more sensitive to shifting temperature and water‐availability regimes than other mammals, providing an ‘early warning’ of their diverse impacts on wild populations. However, regional responses will be mediated by the degree to which physiological traits evolve to match local environmental conditions—a gap that Farrand et al. begin to fill.

Given its high detectability, unique life‐history strategy, and charisma, *O. princeps* (Figure 1b) has proven to be a valuable model organism across many disciplines of ecology and evolutionary biology, including metapopulation theory, island biogeography, and multi‐scale extinction and stepping‐stone dynamics. For this reason alone, the article is likely to garner attention from a wide audience. Multiple other strengths point to its potential long‐term impact. In one of the first range‐wide assessments of local adaptation in *O. princeps* , Farrand et al. provide evidence that can contribute to discussions around the establishment of Evolutionarily Significant Units (ESUs; sensu Funk et al. 2012) for *O. princeps* . Combining dense genomic sampling with multiple tests for selection and a priori hypotheses of the molecular pathways and candidate genes relevant to fitness in cold, high‐elevation, resource‐poor environments, their work is rigorous and thorough. We were particularly struck by the authors' commitment to integrating ecology, physiology and nutritional ecology, biogeography, and climatology. Farrand et al.'s deep knowledge of pika biology is a major asset, here helping to develop a mechanistic understanding of genotype‐environment associations for this climate‐sensitive species.

As thermoclines retract upslope and the montane habitats of this philopatric species shrink and become increasingly isolated, several avenues of future research are likely to be productive. Whole‐genome resequencing data from the six clades sampled here may pinpoint additional outlier loci or structural variants and shed light on the genetic architecture of local adaptation. Phylogenetic reconstruction could help identify the origin of adaptive alleles shared across clade boundaries and distinguish repeated evolution from introgression. Particularly exciting to us is the possibility of integrating microclimate data from within each clade into Farrand et al.'s overall framework. These data could also provide fine‐grained insights into the environmental conditions governing climatic suitability at individually relevant scales, informing future estimates of the strength of selection on physiological tolerances and helping to clarify the relative importance of phenotypic plasticity versus heritable change. We predict they will be especially useful for understanding the role of water‐availability gradients in shaping functional genomic variation in pikas. For example, part of the Sierra Nevada (SN) lineage—highly divergent in allele frequencies at several loci (figure 3 in Farrand et al.)—is among the driest regions occupied by *O. princeps* . Given that aridity appears to be a major abiotic niche axis for pikas and other similar species (Erb et al. 2011; Johnston et al. 2019), additional investigation of the genomic basis of adaptation to water‐stress pathways is warranted.

Lastly, we point to the urgent need to investigate fine‐scale genetic structure within subregions of *O. princeps'* range. Pika populations on ‘sky islands’ in the Great Basin—or in the upper reaches of the Jemez, Sangre de Cristo, and San Juan Mountains in northern New Mexico—have likely been isolated from one another for thousands to tens of thousands of years. If so, drift and selection have likely acted on each in the absence of gene flow, providing a high‐mountain analogue to the multiple conservation units in salmonids frequently found in different drainages or sometimes even different runs in the same river system (e.g., Xuereb et al. 2022). To conserve evolutionary potential and unique adaptive alleles, formal recognition of units that better approximate the shallow timescales of management, conservation, and climate‐adaptation actions will be necessary to ensure the continued survival of species such as American pikas in the face of ongoing global change.^1^

## Author Contributions

All authors contributed to the writing and revising of the manuscript, following EAB's creation of a full initial draft. Contextualization within the broader pika literature reflects EAB's research experience with *O. princeps* since 1994, and EO's since 2023. Contextualization within the broader genomics and molecular‐ecology literatures reflects EBL's and EO's expertise.

## Funding

The authors have nothing to report.

## Conflicts of Interest

The authors declare no conflicts of interest.

## Linked Articles

This article is linked to Farrand et al. paper. To view this article, visit https://doi.org/10.1111/mec.17557.

## Data Availability

The article does not contain any novel data, but is instead a synthetic review of Farrand et al. (this issue).

## Endnotes

^1^
Any use of trade, firm or product names is for descriptive purposes only and does not imply endorsement by the US Government.
